# Phytochemical Screening and Antioxidant Activity of Seven Native Species Growing in the Forests of Southern Chilean Patagonia

**DOI:** 10.3390/molecules26216722

**Published:** 2021-11-06

**Authors:** Merly de Armas-Ricard, Francisco Quinán-Cárdenas, Harold Sanhueza, Rodrigo Pérez-Vidal, Cristina Mayorga-Lobos, Oney Ramírez-Rodríguez

**Affiliations:** 1 Laboratory of Chemistry and Biochemistry, Campus Lillo, University of Aysén. Eusebio Lillo 667, Coyhaique 5951537, Chile; fcob.quinan@gmail.com (F.Q.-C.); harold.sanhueza@uaysen.cl (H.S.); rodrigo.perez@alumnos.uaysen.cl (R.P.-V.); cristina.mayorgalobos@gmail.com (C.M.-L.); 2 Campus Patagonia, Universidad Austral de Chile, Camino a Coyhaique Alto Km. 4, Coyhaique 5950000, Chile; 3 Faculty of Sciences, University of Chile, Las Palmeras 3425, Santiago 7800003, Chile; 4 Faculty of Chemical and Pharmaceutical Sciences, University of Chile, Santos Dumont 964, Santiago 8380494, Chile

**Keywords:** antioxidant capacity, sun protector factor, polyphenols, α-glucosidase inhibitors, tyrosinase inhibitors, *Nothofagus*, *Berberis microphylla*, *Aristotelia chilensis*

## Abstract

The genus *Nothofagus* is one of the most abundant in the subantarctic Patagonian forests. Five species inhabit these ecosystems, three evergreen (*Nothofagus betuloides*, *Nothofagus dombeyi*, and *Nothofagus nitida*) and two deciduous (*Nothofagus pumilio* and *Nothofagus antarctica*). This is the first report on the levels of secondary metabolites and the antioxidant capacity of Patagonian tree species growing in natural environments. The aim of this work was to carry out a phytochemical screening, to determine the antioxidant capacity, the sun protection factor, and the α-glucosidase and tyrosinase inhibitory activity of foliar extracts of the five previous species. Besides, *Aristotelia chilensis* and *Berberis microphylla*, two species of Patagonian shrubs growing in the same forests, were used as reference. *N. dombeyi* was the *Nothofagus* with the best antioxidant capacity. *B. microphylla* differed from all studied species. Moreover, the *Nothofagus* was split into two groups. *N. betuloides* and *N. dombeyi* are the most similar species to *A. chilensis*. The α-glucosidase was completely inhibited by all studied extracts. Furthermore, *N. antarctica*, *N.* *pumilio*, and *N. nitida* inhibited about 70% of the tyrosinase activity. All the results found in this study for the species of the genus *Nothofagus* support further research on their potential beneficial properties for human health.

## 1. Introduction

Patagonia is a geographical region located at the southern end of South America. This region is the closest to Antarctica, being the only landmass in these southern latitudes. It is characterized by having a pristine nature, with lakes, glaciers, fjords, steppes, channels, and forests. Eastern Patagonia, located east of the Andes Mountain and known as Argentine Patagonia, is mostly steppe. Western Patagonia or Chilean Patagonia has a great variety of climates, varying from the rainy on the Pacific coast to the steppes on the border with Argentina. This variation in rainfall results in two different forest formations, an evergreen forest on the Pacific Ocean coast and a deciduous forest inland. Most of the Patagonian subantarctic forest is in the Aysén Region, covering more than 5 million hectares with a high degree of biodiversity and endemism [1,2,3]. A little less than half of its surface (41%) is included within the system of protected wild areas (National Parks and National Reserves).

There are several species of shrubs and trees abounding in the Chilean Patagonia subantarctic forests. Among the most common shrubs are different species of the genus *Berberis*, such as michay (*Berberis darwinii*) and calafate (*Berberis microphylla*). These species, maqui (*Aristotelia chilensis*) and some others, make up the so-called forest berries. There are many studies on various properties of forest berries. Among them, the antioxidant properties of their fruits and their benefits for humans have been studied [4,5,6,7,8,9,10].

The most common tree species belong to the genus *Nothofagus*. They are known as Southern hemisphere beech trees, being common in South America, Australia, and New Zealand [11]. There are 10 species throughout its entire distribution in Chile. Among the most common species in the evergreen forests are the dombey’s beech (*Nothofagus dombeyi*) and the Magellan’s beech (*Nothofagus betuloides*), while the most abundant deciduous species are lenga beech (*Nothofagus pumilio*) and ñire (*Nothofagus antarctica*). These two deciduous species are the most widely distributed species of *Nothofagus* genus, covering almost 20 degrees latitude (2200 km). There are many studies on the ecology of *Nothofagus* forest, morphological and phenological differences on an altitudinal gradient, genetic variations, intraspecific trait variation, climate–growth relationship, colonization routes after glacial periods, adaptative and plastic responses, dendrochronology, and forest ecology of this species throughout its distribution, both in Chile and Argentina [12,13,14,15,16,17,18,19,20,21,22,23,24]. However, studies on the composition of foliar secondary metabolites of *Nothofagus* species are very scarce and are related to the response to biotic stress (attack by herbivorous insects) [25,26]. There are no reports on the antioxidant capacity, nor the action of these species extracts, on the enzymatic activities or other properties associated with human health. In this work, a foliar phytochemical analysis and the antioxidant capacity of five *Nothofagus* species are reported. These data are compared with those obtained for *A.* *chilensis* and *B. microphylla* foliar extracts, growing in the same environment.

Polyphenols are a broad family of organic compounds characterized by having one or more hydroxyl groups bounded to a benzene ring. They are subdivided into several families, such as flavonoids, coumarins, stilbenes, phenolic acids C6-C3 and C6-C1, anthocyanins, phenylpropanoids, and so on. All of them have diverse biological functions in plants and expressed antioxidant capacity [27,28,29,30,31]. Phenolic compounds are considered natural antioxidants. An increase in their biosynthesis has been reported under conditions of severe stress, when the activity of antioxidant enzymes decreases, considered the first line of defense against ROS [32,33,34]. Additionally, it has been reported that these compounds accumulate in response to stress conditions owing to temperature, water availability, salinity, heavy metals, herbicides, and high levels of ultraviolet radiation, contributing to the plants adaptation to unfavorable environments [27,30,34,35,36,37,38]. These compounds have several properties of interest to today’s society. They are fundamental constituents of fruits and vegetables, considered functional foods. Moreover, they can be used as nutraceutical additives and food preservatives. In addition, they have antimicrobial, antitumor, and antidiabetic properties, among others [39,40,41,42,43,44,45,46].

The ozone layer deterioration has resulted in an increase in ultraviolet radiation on the Earth’s surface. This ultraviolet radiation is harmful to humans because it causes DNA damage with consequences ranging from mild skin changes to skin cancer. For this reason, a set of dermo-cosmetic products has been developed, which are characterized by having a certain sun protection factor. There is a worldwide trend towards the use of natural extracts because, in general, they are less toxic, and when mixed in the right amounts, they produce positive effects for human health [47,48,49]. The sun protection factor (SPF) is an index used to characterize all substances or mixtures capable of acting as sunscreens. The spectrophotometric method reported by Mansur et al. [50] is widely used for this purpose [51,52,53].

The skin is the largest organ in the human body, and it becomes diseased, just like any other part of the body. Just under 2% of diseases in the world are attributed to the skin. In the United States, according to The American Academy of Dermatology Association, about 25% of the population suffers from some skin disease [54]. The human hair, skin, and eyes colors are due to a substance called melanin. This compound is synthesized from the amino acid L-tyrosine. Tyrosinase is a copper-containing glycoprotein. This enzyme is the rate-limiting for melanin biosynthesis pathway [55]. It catalyzes the L-tyrosine oxidation to L-DOPA, and its subsequent oxidation to DOPA quinone (dopachrome). It is a highly conserved protein present in plants, animals, and bacteria [56]. Irregular skin hyperpigmentation is caused by abnormal distribution or excessive production of melanin. For this reason, tyrosinase inhibitors are being sought to improve or prevent hyperpigmentary disorders, such as age spots and melasma [55]. The medical and cosmetic industries are interested in finding new tyrosinase inhibitors to treat and prevent skin disorders.

Diabetes mellitus is a metabolic disease. There are two types of it, 1 and 2. The second is the most common, manifesting itself mainly in adults. It appears when the body does not synthesize enough insulin, or the cells become resistant to that hormone. This type of diabetes has dramatically increased prevalence in the last 30 years. It is strongly associated with obesity and sedentary lifestyles. This chronic disease is characterized by a high level of glucose in the blood, and it causes damage to various organs, such as the kidneys and the heart, among others. It is suffered by about 422 million people in the world, and around 1.6 million deaths per year are attributed to it [57].

Currently, this disease is incurable. Diabetic patients must have strict control over their carbohydrate intake. For this reason, most drugs that control diabetes are aimed at inhibiting glycosidases, the digestive enzymes that hydrolyze carbohydrates. Among them, the most abundant in humans are salivary and pancreatic amylases, sucrase, and maltase. All of them are α-glucosidases. The inhibition of these enzymes means that carbohydrates cannot be degraded to monosaccharides in the small intestine and, therefore, the amount absorbed is minimized, controlling the blood glucose concentration. Some drugs, such as acarbose, have been successful [58]. Currently, there is a trend to search for natural products and extracts with α-glucosidase inhibitory activity because, in general, they are less toxic than synthetic drugs [59,60,61,62,63,64,65,66].

There are few field data on the composition of foliar secondary metabolites in wild tree species in their natural habitats in other latitudes, but in southern Patagonia, they do not exist. There are also just few reports on antioxidant activities measured by different methods in forests, in contrast to crop plants and their fruits. The antioxidant capacity of trees and their composition of secondary metabolites are closely related to the resistance of these species to environmental stressors caused by climate change. For this reason, shedding light on these aspects contributes to the knowledge about the survival of these ecosystems to the new climate change conditions. Besides, species showing high antioxidant capacity can be used as a source to obtain new compounds and/or extracts with multiple and potential applications in health, as well as in the pharmaceutical and nutraceutical industries, opening opportunities for new products from forests. These forest products, unlike classic forestry, are sustainable over time and add value to these ecosystems.

The absence of these studies has kept the door closed to a set of potential novel applications for these subantarctic forests. Many medicinal herbs are used by the inhabitants of the region, transmitting popular knowledge from generation to generation, but there are no studies or uses attributed to the tree species of the South American subantarctic forests. The aim of this work was to carry out a phytochemical screening and the foliar antioxidant capacity determination of seven native Patagonian species (*Nothofagus betuloides*, *Nothofagus dombeyi*, *Nothofagus nitida*, *Nothofagus antarctica*, *Nothofagus pumilio*, *Berberis microphylla*, and *Aristotelia chilensis*). Besides, the antidiabetic potential, the sun protection factor, and the tyrosinase inhibitory activity were determined. This research contributes to providing a scientific basis for the use of Patagonian species foliar extracts and to shed light on their properties and potential applications.

## 2. Results and Discussion

The most abundant tree species in the Aysén region belong to the genus *Nothofagus*. Three of them are evergreen (*N. betuloides*, *N. nitida,* and *N. dombeyi*) and two are deciduous (*N. antarctica* and *N. pumilio*). All, except *N. nitida*, are part of the forest canopy. Among the most common shrubs in these forests are berries, such as *Aristotelia chilensis* and *Berberis microphylla*.

### 2.1. Phytochemical Screeening

**Figure 1:**
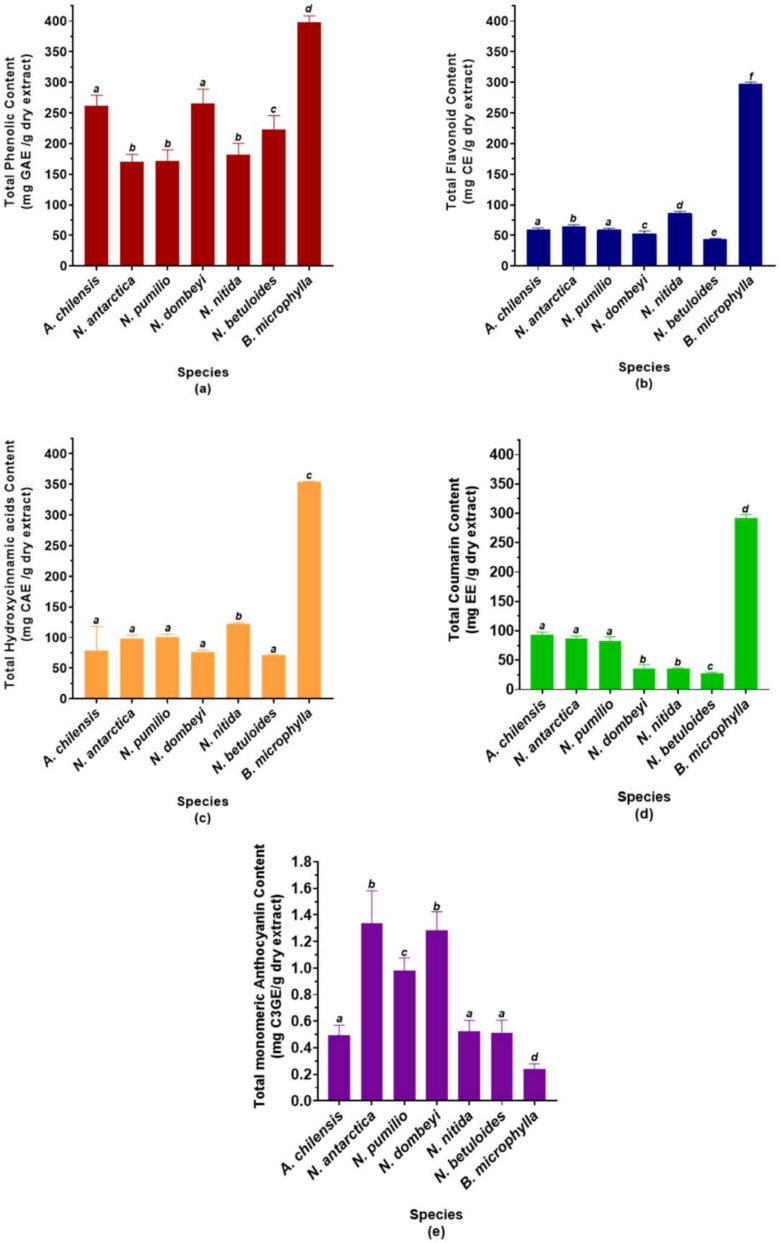
Phytochemical screening of the studied species. Data are expressed as means ± SD (n = 9). (**a**) Total phenolic content, expressed as gallic acid equivalent (GAE) per gram of dry extract. (**b**) Total flavonoid content, expressed as catechin equivalent (CE) per gram of dry extract. (**c**) Total hydroxycinnamic acids derivatives content, expressed as caffeic acid equivalents (CAEs) per gram of dry extract. (**d**) Total coumarin content, expressed as esculetin equivalents (EEs) per gram of dry extract. (**e**) Total monomeric anthocyanin content, expressed as cyanidin 3-*O*-glucoside per gram of dry extract. The different letters on the bars indicate significant differences with an average value of *p* < 0.05, Tuckey’s test.

The phytochemical analysis of the studied species is shown in Figure 1. Calafate (*Berberis microphylla*) and maqui (*Aristotelia chilensis*) are two species of recognized antioxidant power in their fruits. Calafate leaves show the highest content of phenolic compounds (Figure 1a). Notice that there are no statistically significant differences between the TPC of *A. chilensis* and *N. dombeyi*. Among the *Nothofagus* species, the deciduous and *N. nitida* show the lowest TPC, while *N. betuloides* and *N. dombeyi* have the highest level of polyphenols.

In contrast to the total phenolic content, the total flavonoid content of the studied species is more similar, except for *B. microphylla* (Figure 1b). Calafate leaves show between three and six times more flavonoids than the other species. Additionally, and unlike TPC, it is observed that *N. nitida* is the *Nothogafus* species with the highest flavonoid content. *N. dombeyi* and *N. betuloides* show the lowest content of these metabolites. The other species of *Nothofagus* and *A. chilensis* show a similar total flavonoid content.

*Berberis microphylla* leaves also show the highest hydroxycinnamic acid derivatives content (Figure 1c). Moreover, *N. nitida* is the *Nothofagus* species with the highest THC. The other species of this genus show total levels of hydroxycinnamic acids derivatives statistically similar to the *A. chilensis*.

*B. microphylla* leaves show the highest total coumarin content (Figure 1d), being between 3 and 10 times higher than the other species. The coumarin level of the deciduous *Nothofagus* is more than twice that of the evergreen, showing no statistically significant differences with *A. chilensis*. Furthermore, evergreen species show low levels of coumarins, with *N. betuloides* dispaying the least.

Unlike everything previously observed, we can see that *B. microphylla* leaves show the lowest anthocyanin content (Figure 1e). *N. antarctica*, *N. dombeyi,* and *N. pumilio* are the *Nothofagus* species with the highest anthocyanin content. The other *Nothofagus* species show statistically similar levels to *A. chilensis*, being between two and three times lower than *N. antarctica*, *N. dombeyi*, and *N. pumulio*.

It is observed that the polyphenol content of the *Nothofagus* evergreen species is significantly higher than the deciduous species, except for *N. nitida*. Winter frosts have been described as the main cause of mortality in *N. dombeyi* and *N. nitida* seedlings [67]. Additionally, it has been reported that *N. betuloides* shows a positive correlation between growth and temperatures in early summer (December–January) [68]. This suggests that low temperatures could be a stressor for these evergreen forest species. The samples for this study were taken in October, at the beginning of the austral spring, and they could reflect the increase in TPC because of a stress condition due to the winter just ended. Deciduous species probably have a lower TPC level than evergreen species because their metabolism is minimized during winter, and they are not subjected to this prior stress factor. It is important to note that *N. dombeyi* and *A. chilensis* do not show significant differences in TPC. This result opens the door to future studies and new applications for this species from the Patagonian evergreen forest. Currently, secondary metabolites studies and the seasonal composition variation of these species are being carried out.

### 2.2. Antioxidant Capacity

**Figure 2:**
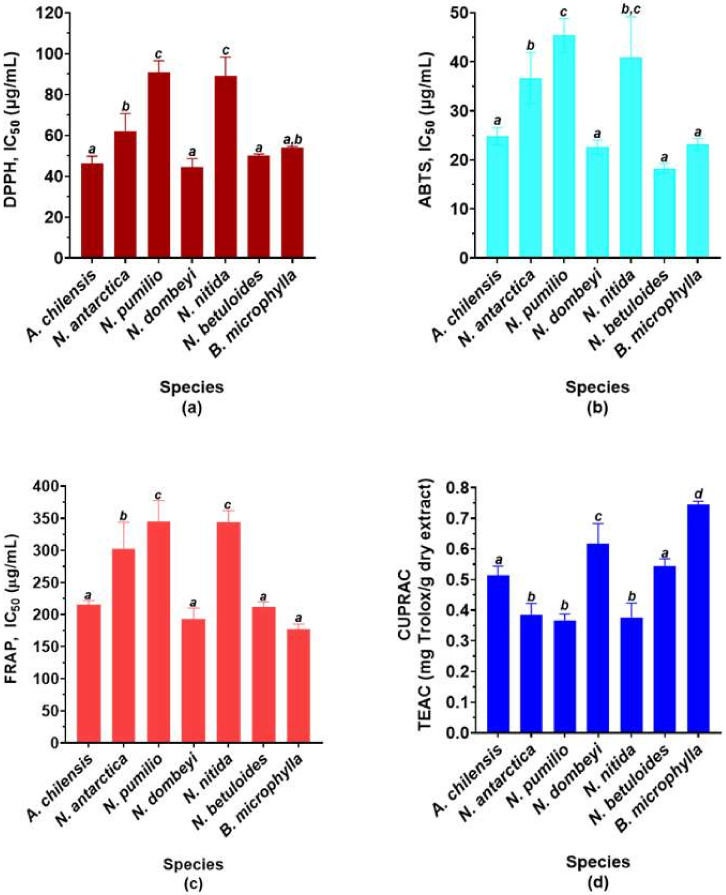
Antioxidant capacity of the studied species. Data are expressed as means ± SD (n = 9). (**a**) DPPH radical scavenging activity (IC_50_ μg/mL). (**b**) ABTS radical scavenging activity (IC_50_ μg/mL). (**c**) FRAP (IC_50_ μg/mL). (**d**) CUPRAC expressed as equivalents of trolox (TEAC) per gram of dry extract. The different letters on the bars indicate significant differences with an average value of *p* < 0.05, Tuckey’s test.

The antioxidant capacity of the species is shown in Figure 2. It shows the ability of the extracts to eliminate free radicals, and thus prevent oxidative damage (ABTS and DPPH radical scavenging activities). Besides, it shows the ability to reduce oxidized species, and thus the prevention and reversion of oxidative damage (ferric and cupric reducing power, FRAP and CUPRAC, respectively).

As expected, both *A. chilensis* and *B. microphylla* show high DPPH and ABTS free radical scavenging activities, being statistically similar (Figure 2a,b). Surprisingly, *N. dombeyi* and *N. betuloides* have an IC_50_ value statistically similar to calafate and maqui in both assays. However, *N. pumilio* and *N. nitida* show similar IC_50_ values to each other, but higher than the other species of the genus. Moreover, *N. antarctica* shows a lower ABTS radical scavenging activity, being similar to *N. nitida*.

The ferric reducing antioxidant power (FRAP, Figure 2c) of the species shows a behavior like the ABTS free radical scavenging activity. However, the cupric reducing antioxidant capacity (CUPRAC, Figure 2d) does not behave in the same way in several cases. *Nothofagus dombeyi* and *Berberis microphylla* are the two species with the highest copper (II) ion reduction capacity, with the highest being in the latter. *A. chilensis* and *N. betuloides* show values significantly similar and lower than the previous two. Finally, the other three species of *Nothofagus* have statistically similar values.

Considering all the previous results, we can conclude that *N. dombeyi* and *N. betuloides* are the *Nothofagus* species with the best antioxidant capacity, the first being the best of all.

The antioxidant capacity of *Aristotelia chilensis* and *Berberis microphylla* fruits is widely documented [4,5,6,7,8,9,10]. However, there are few reports of the antioxidant capacity of foliar extracts of *Aristolelia chilensis* [69,70,71] measured as DPPH free radical scavenging activity. Céspedes et al. [69] reported IC_50_ values between 3.5 and 98.2 μg/mL. The extracts were obtained by fractionation with different solvents from an initial alcoholic extract. The highest antioxidant capacity was obtained using ethyl acetate for the extraction. Rubilar et al. [70] reported an IC_50_ value of 8 µg/mL for a hydroalcoholic extract (50% *v/v*). González-Villagra et al. [71] reported the DPPH free radical scavenging activity in maqui leaves under water stress ranges between 25 and 30 mg of Trolox/g of dry weight. All these reports are made with maqui plants grown more than 1300 km north of central Patagonia. Furthermore, all these extracts were obtained using different solvents and extraction conditions. This makes comparison to our data difficult. Finally, these authors did not find reports of antioxidant capacity in *Berberis microphylla* leaves. There are no reports of the antioxidant capacity of the Patagonian *Nothofagus*. For this reason, in this work, we compare the antioxidant capacity of these tree species, measured in various ways, to *A. chilensis* and *B. microphylla*.

The obtained results in this work open new use options for the *Nothofagus* and *B. microphylla* foliar extracts. There are recent reports [72] of the improvement in the stability of edible oils when foliar extracts of maqui are added to them. This shows the possibility of using natural additives as a healthier alternative to the use of synthetic additives in food preservation.

### 2.3. Correlation and Principal Component Analyses

**Figure 3:**
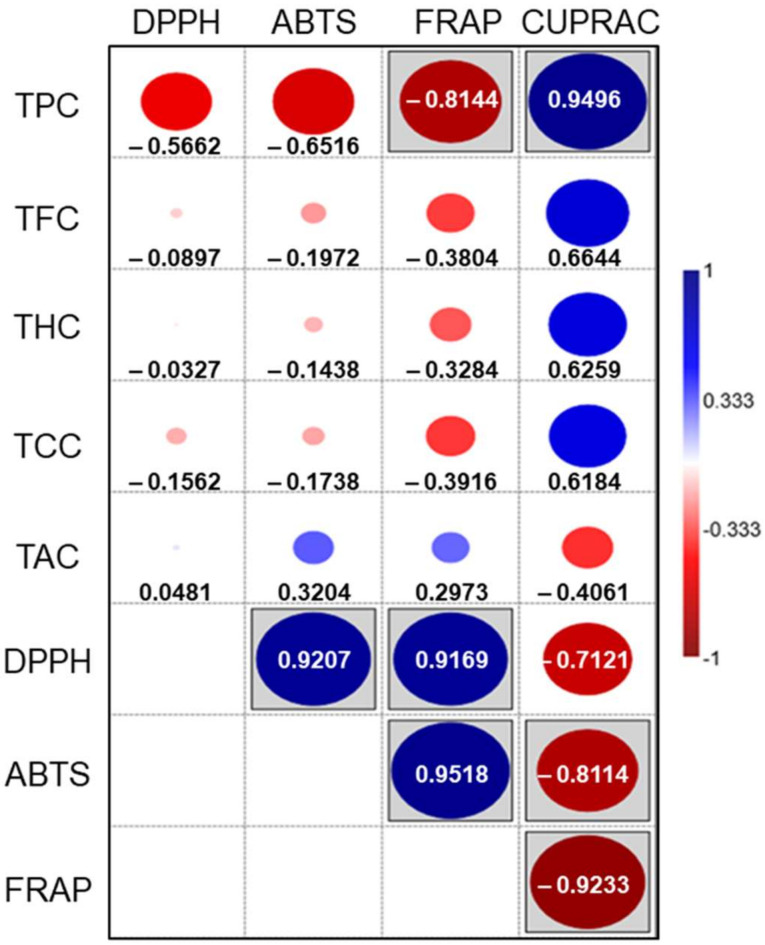
Pearson’s correlation between secondary metabolites and antioxidant capacities. The blue color represents positive correlations. The red color represents negative correlations. Boxed figures are statistically significant correlations (*p* < 0.05). The circle diameter is proportional to the Pearson’s correlation coefficient (r), specified in each case with the number.

The Pearson correlation coefficient values and the correlations’ statistical significance are shown in Figure 3. As expected, negative correlations are observed between most of the metabolites and antioxidant activities given as IC_50_ (DPPH, ABTS, and FRAP). Statistically significant correlations are observed between the TPC and the metal reduction tests, and among most antioxidant capacity assays.

The highest correlation coefficients between metabolites and antioxidant activities are observed for TPC. Individualized polyphenol subgroups do not correlate significantly with antioxidant activities. This suggests that their antioxidant activity, as a family of polyphenols, could be due to synergistic effects among all subgroups. Unlike the above, the correlations among all antioxidant activities are statistically significant, showing high Pearson correlation coefficients, except for DPPH–CUPRAC.

It is interesting to highlight the good correlation between free radical scavenging activities and iron reducing power, suggesting the capacity of extracts to keep cells in a reduced state. The combined effect of the free radical scavenging activities and the ability to reverse oxidative damage of these foliar extracts supports further research on their potential beneficial properties to human health.

**Figure 4:**
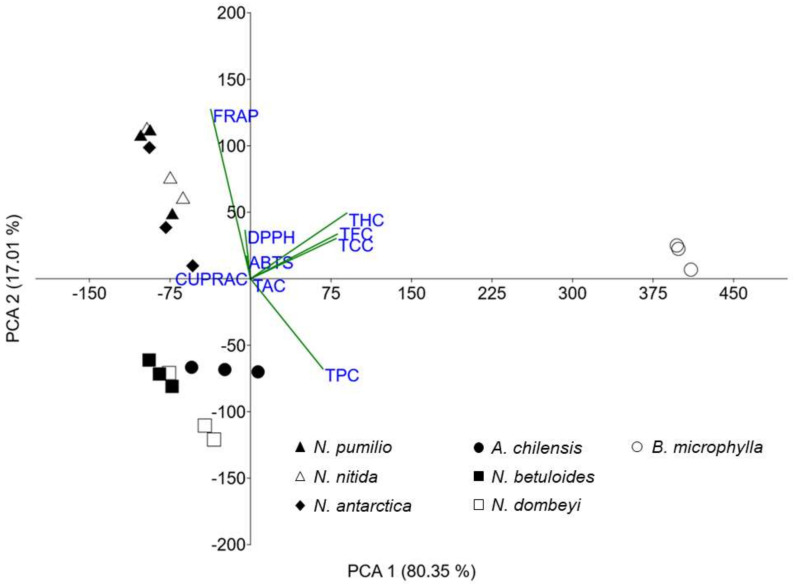
Biplot from the principal component analysis model between all studied variables.

To determine the influence of all the studied variables on the antioxidant behavior, a principal component analysis (PCA) was performed (Figure 4). The loading vectors values are shown in Table S1 (Supplementary Materials).

The *X*-axis (first component) explains 80.35% of the variance, and the separation in this axis is mainly due to THC (0.55), TFC (0.49), TCC (0.49), and TPC (0.41). The *Y*-axis (second component) explains 17.01% of the variance, and the separation in this axis is mostly due to FRAP (0.78) and TPC (−0.41). Between the first two main components, 97.36% of the total variance is explained; therefore, multivariate analysis is reliable and it does not require the use of another component.

In Figure 4, we can see that the *B. microphylla* foliar extract shows a different antioxidant capacity and a composition of metabolites from the other studied species. The *Nothofagus* are divided into two groups. *N. pumilio*, *N. nitida*, and *N. antarctica* are in one, while *N. betuloides* and *N. dombeyi*, together with *A. chilensis*, are in the other. This principal component analysis shows the latter *Nothofagus* have antioxidant properties similar to *A. chilensis*. This opens up a set of potential applications for foliar extracts of these species.

The analysis of similarity (ANOSIM, Table 1) yielded a value of *p* = 0.0001 among the compared groups (species), suggesting that all of them are statistically different. The values of R, with three exceptions, are greater than 0.7778, and 1 in most cases. This shows there is dissimilarity among all species. The two species with the greatest similarity are *N. pumilio* and *N. antarctica* (R = 0.037). *N. dombeyi* and *N. betuloides* (R = 0.2963), as well as *N. nitida* and *N. pumilio* (R = 0.5926), also show R values that indicate a more similar antioxidant behavior.

The similarity percentages breakdown procedure (SIMPER) shows the overall average dissimilarity (OAD) values (Table S2, Supplementary Materials). All comparisons between the extracts of any species and the leaf extract of *B. microphylla* show an OAD greater than 37.479. Comparisons between all other species show an OAD less than 25.271. The comparison of *N. betuloides*–*N. dombeyi* (7.339) and *N. pumilio*–*N. antarctica* (7.564) confirms that these are the pairs of species with the most similar behavior. Furthermore, the similar behavior of *A. chilensis* with *N. dombeyi* (9.792) and *N. betuloides* (11.135) is shown. In general, the greatest contribution to dissimilarity is given by the FRAP value.

### 2.4. Sun Protector Factor (SPF)

The SPF values are shown in Table 2. The highest value of the sun protection factor per milligram of dry extract is exhibited by the *Berberis microphylla* leaf extract, followed by the *Aristotelia chilensis* leaf extract. Besides, *N. betuloides* and *N. dombeyi* exhibit similar, but lower values than *A. chilensis*. The SPF values of *N. antarctica* and *N. pumilio* are lower than the previous ones, while the SPF of *N. nitida* is the lowest of all.

The natural extracts benefits for skin care are increasingly recognized because synthetical sunscreens may have adverse effects on the skin, such as dermatitis, irritation, sensitivity, and so on [73]. Four protection levels have been proposed for sunscreens. Maximum protection level, for substances with SPF greater than 50. High protection level, for substances with SPF between 30 and 50. Medium protection level, for substances with SPF between 15 and 30. Low protection level, if the SPF value is between 2 and 15 [74]. Taking into account the above, our extracts can be classified as medium and low level for concentrations around 200 μg/mL.

Plant extracts with photoprotective activity have been reported before [73,75,76,77,78]. These extracts have been obtained from different plant organs and their composition is diverse. Polyphenols predominate in them [79,80]. The SPF values of most of the substances and extracts reported are determined at concentrations between 1 and 2 mg/mL [73,76]. Our results suggest that the foliar extracts of *B. microphylla*, *A. chilensis*, *N. dombeyi*, *N. antarctica*, and *N. betuloides* have potential use as sunscreens, because, even at low concentrations, a protective effect is detected.

### 2.5. Glucosidase and Tyrosinase Inhibitory Activity

The enzymatic inhibitory activities of the extracts are shown in Table 3. All extracts were tested at about 100 µg/mL, while acarbose was tested at about 600 μg/mL. Most of the studied extracts show excellent α-glucosidase inhibition, even better than acarbose, despite being six times more diluted. It is noteworthy that the five species of the genus *Nothofagus* totally inhibit the enzyme. The inhibition percentage values found to *A. chilensis* and *B. microphylla* foliar extracts are also exceptionally good, although slightly lower.

Inhibition of carbohydrate-degrading enzymes in the human digestive tract is a good strategy to control the blood glucose level. Substances or mixtures having this property have potential use as antidiabetic drugs, and functional foods. There are several reports of the α-glucosidase inhibitory activity of maqui and calafate fruit extracts [70,81]. These authors did not find reports on foliar extracts of species of the genus *Nothofagus* having this activity. These results open new options for potential antidiabetic uses of the foliar extracts of the *Nothofagus* species. Additional studies are necessary to calculate the IC_50_ of these extracts and the mechanisms of inhibition of the enzyme.

Considering that the tested concentrations were not exactly the same, some general discussions on the tyrosinase inhibition by these extracts can be obtained. *N. antarctica* exhibited the highest percentage of tyrosinase inhibition with the lowest concentration, suggesting that it is the extract with the best tyrosinase inhibitory activity. *N. nitida* and *N. pumilio* extracts showed very similar inhibition percentages with different concentrations. This suggests that *N. nitida* extract could have greater activity than the *N. pumilio* extract. The *N. dombeyi*, *N. nitida*, and *A. chilensis* extracts were tested at very similar concentrations. They showed a great difference in their inhibitory activity. This suggests that the *N. nitida* extract could be the most active of these three. Furthermore, the *B. microphylla* extract showed greater activity than the *A. chilensis*, *N. betuloides*, and *N. dombeyi* extracts, despite being more diluted. This suggests that this extract has better enzyme inhibitory activity. Finally, the *N. betuloides* extract is the second most concentrated and showed the lowest inhibitory activity. This suggests that this species could have the lowest tyrosinase inhibitory activity. Some authors have reported the IC_50_ for kojic acid (30.6 μM [82], 14.2 μg/mL [69], 59.8 μM [83], and 35.62 μg/mL [84]). Most natural extracts are reported to have lower tyrosinase inhibitory activity than kojic acid [84,85]. However, our extracts’ inhibitory activity is greater than arbutin, a drug used as a tyrosinase inhibitor (arbutin, IC_50_ 1.36–7.62 mg/mL [84,86,87]). These results are promising, considering that we are working with complex composition extracts, not with pure compounds.

There are some reports of tyrosinase inhibitory activity by maqui fruits (IC_50_ 5.31 μg/mL) [84] and maqui leaves (IC_50_ 8.4 μg/mL, ethyl acetate fraction, IC_50_ 39.8 μg/mL, aqueous residue, with respect to L-DOPA) [69] obtained from regions located further north of Chile, not from Patagonia. There are no reports of IC_50_ of Chilean Patagonian maqui or any species of *Nothofagus* from anywhere. Further studies are necessary to fractionate and/or to determine the IC_50_ for these extracts. The found that the results for these three *Nothofagus* species open a new study field for skin diseases’ treatment using these foliar extracts.

## 3. Materials and Methods

### 3.1. Chemicals and Equipment

All reagents, enzymes, and solvents were purchased from Merck and Sigma-Aldrich (Chile). All quantitative determinations were carried out using a Biotek Epoch 2 microplate spectrophotometer in NUNC or Falcon 96-well plates.

### 3.2. Species under Study and Collection of Plant Material

The studied species are some of the most common tree species in Patagonian forests and two shrub species. The tree species are *Nothofagus dombeyi* (Dombey’s beech, in Spanish coigüe común), *Nothofagus betuloides* (Magellan’s beech, in Spanish guindo or coigüe de Magallanes), *Nothofagus nitida* (Chiloé beech, in Spanish coigüe de Chiloé), *Nothofagus pumilio* (lenga beech, in Spanish lenga), and *Nothogafus antarctica* (Antarctic beech, in Spanish ñire or ñirre). The shrubs are the *Berberis microphylla* (box-leaved barberry or Magellan barberry, in Spanish calafate) and *Aristotelia chilensis* (Chilean wineberry, in Spanish maqui). All are evergreen species, except *N. pumulio* and *N. antarctica*. Samples of the collected specimens were deposited in the herbarium of the Laboratory of Chemistry and Biochemistry at the University of Aysén, in Chilean Patagonia (Figure S1).

*Nothofagus pumilio* was sampled at Cerro Castillo National Park (46°4′7.60″ S, 72°1′12.67″ O, 1060 m.a.s.l.). *Nothofagus betuloides*, *Nothofagus dombeyi*, *Nothofagus nitida,* and *Aristotelia chilensis* were sampled at Simpson River National Reserve (45°28′3.99” S, 72°18′34.58″ O, 100 m.a.s.l.). *Nothofagus antarctica* and *Berberis microphylla* were sampled at private property in Vista Hermosa (45° 57′35.43″ S, 71° 46′43.83″ O, 688 m.a.s.l.).

Foliar samples were obtained from three plots at each study area. The plots were 10 m by 10 m separated between 30 and 40 m from each other. The leaves were collected from at least six individuals within each plot. They were located three meters high (trees), directly exposed to the sunlight. All the trees were approximately the same phenological age (chest height circumference between 70 and 90 cm), and visually healthy. All samples were collected in the spring (October–November 2019). Few leaves (approximately 150–200 leaves) were collected from each tree or shrub, in order to avoid excessive defoliation. No more than 10 flowers were collected from each individual.

### 3.3. Phytochemical Analysis

To determine the antioxidant capacity and the phytochemical analysis, extracts were prepared from a dry material. Briefly, the leaves or flowers were dried at 40 °C in a stove and powdered. Extracts were prepared by mixing the powdered material and ethanol (1:6, *w/v*) at 25 °C, shaking gently for 18 h in the dark. The solvent was filtered and evaporated in vacuo. The extracts obtained were stored at −20 °C for subsequent analysis. Working solutions were prepared by dissolving the dry extract in ethanol at a final concentration of 1 mg/mL.

#### 3.3.1. Determination of Total Phenolic Content (TPC)

The determination of the total phenolic content (TPC) was based on the method described by Ainsworth et al. [88] and Bridi et al. [89], with some modifications. Briefly, the mixture of 10% (*v/v*) Folin–Ciocalteu reagent and leaves extract dissolved in ethanol was incubated for 5 min at 25 °C. After that, 350 mM sodium carbonate was added. The final mixture was incubated for 2 h, and the absorbance was determined at 765 nm. A calibration curve of gallic acid was made using gallic acid solutions (15–75 µg/mL). The results were expressed as milligrams of gallic acid equivalents (GAEs) per gram of dry extract (mg GAE/g SD). Values are reported as means ± standard deviations of four independent determinations.

#### 3.3.2. Determination of Total Flavonoid Content (TFC)

Total flavonoid content (TFC) was determined using the method described by Dewanto et al. [90], with some modifications. Briefly, the leaves’ ethanolic extract, or (+)-catechin standard solution, or ethanol (blank solution) was added to each of the 96-well microplates, followed by sodium nitrite solution (4.54 mg/mL). The mixture was incubated for 5 min at 25 °C. Then, the aluminum chloride solution (50 g/L) was added, and the mixture was incubated for 6 min. After that, the sodium hydroxide solution (0.5 M) was added. The plate was stirred vigorously for 2 min. The absorbance was measured immediately against the blank at 510 nm using a spectrophotometer. The standard curve was prepared with (+)-catechin (25–100 µg/mL). The results are expressed as means (milligrams of catechin equivalents per gram of dry extract) ± SD of four independent determinations.

#### 3.3.3. Determination of Total Coumarin Content (TCC)

Total coumarin content (TCC) was determined using the method described by Olennikov et al. [91], with modifications using ethanol as solvent. Briefly, 20 mg of dry extract was dissolved in a 20 mL volumetric flask using ethanol as solvent. The sample was diluted (1:10) or (1:5), as appropriate, and the absorbance at 350 nm was measured using a microplate spectrophotometer. An esculetin curve was prepared using concentrations between 1 and 30 μg/mL. The results are expressed as means (milligrams of esculetin equivalents per gram of dry extract) ± SD of four independent determinations.

#### 3.3.4. Determination of Total Hydroxycinnamic Acid Derivatives Content (THC)

Total hydroxycinnamic acids derivatives content was determined using the method described by Matkowski et al. [92], based on the method described by Arnow [93]. Briefly, in each well of a 96-well plate, 40 μL of the stock extract solution was mixed sequentially with 40 μL of 0.5 M hydrochloric acid solution, 40 μL of Arnow’s reagent (10% *w/v* aqueous solution of sodium nitrite and sodium molybdate), 40 μL of sodium hydroxide solution (8.5% w/v), and 40 μL of distilled water. The plate was shaken for 15 s, and the absorbance was measured immediately at 500 nm, using 40 μL of solvent instead of 40 μL of extract as a blank solution. Caffeic acid was used as the reference compound. The standard calibration curve was generated using concentrations between 25 and 325 μg/mL in ethanol. All analyses were performed four times and the data are expressed as mean value ± standard deviation (SD).

### 3.4. Determination of Antioxidant Capacity

#### 3.4.1. DPPH Free Radical Scavenging Activity

The DPPH (2,2-diphenyl-1-picrylhydrazyl) free radical scavenging activity was determined following the method described by Ben Mrid et al. [94] and Hartwig et al. [95], with some modifications. Briefly, the ethanolic extract solutions were mixed with a freshly prepared DPPH solution. The mixture was incubated for 30 min at 25 °C. The absorbance was measured at 517 nm. Trolox was used as a positive control. A DPPH standard curve (from 20 to 140 µM) was made to quantify its remaining concentration in all experiments. All analyses were performed four times and the data are expressed as mean value ± standard deviation (SD). The percentage of DPPH scavenging activity was determined by the following equation:

$$\%\;\mathrm{DPPH}\;\mathrm{Scavenging}\;\mathrm{Activity}=\left(\frac{C{\left(\mathrm{DPPH}\right)}_{\mathrm{BLANK}}-C{\left(\mathrm{DPPH}\right)}_{\mathrm{SAMPLE}}}{C{\left(\mathrm{DPPH}\right)}_{\mathrm{BLANK}}}\right)100$$

The IC_50_ of each extract was determined as the concentration that corresponds to 50% of the DPPH scavenging activity.

#### 3.4.2. ABTS Radical Scavenging Activity

ABTS scavenging activity was determined using the method described by Jimoh et al. [96] and Iauk et al. [97], with some modifications. Briefly, the ABTS radical was generated by mixing equal volumes of ABTS and potassium persulfate solutions. For a 96-well plate, 10 mL of this reagent was prepared. For this, 200 µL of 2.45 mM aqueous potassium persulfate solution was mixed with 200 µL of 7 mM ethanolic solution of ABTS (400 µL total). The mixture was in the dark and at room temperature for 12 to 18 h. Finally, it was diluted with ethanol to a final volume of 10 mL (1:25 ratio), and the absorbance of the resulting solution must be between 0.7 and 0.8.

Then, 100 μL of ethanolic extract, the positive control (BHT ethanolic solution, IC_50_ = 11.57 µg/mL), or the extracting solvent (blank) was mixed with 100 μL freshly prepared ABTS reagent (1:1 *v/v* mix). The mixture was incubated for 6 min at room temperature and absorbance was measured at 734 nm. All analyses were performed four times and the data are expressed as mean value ± standard deviation (SD). The ABTS scavenging activity percentage was determined by the following equation:

$$\%\;\mathrm{ABTS}\;\mathrm{Scavenging}\;\mathrm{Activity}=\left(\frac{A{\left(\mathrm{ABTS}\right)}_{\mathrm{BLANK}}-A{\left(\mathrm{ABTS}\right)}_{\mathrm{SAMPLE}}}{A{\left(\mathrm{ABTS}\right)}_{\mathrm{BLANK}}}\right)100$$

The IC_50_ of each extract was determined as the concentration that corresponds to 50% of the ABTS scavenging activity.

#### 3.4.3. Ferric Reducing Antioxidant Power (FRAP)

FRAP was determined using the method described by Bridi et al. [89] and Iauk et al. [97], with some modifications. Briefly, the FRAP solution was prepared by mixing TPTZ solution (20 mM in hydrochloric acid), iron (III) chloride (20 mM), and acetate buffer (pH 3.6). The extract or the solvent (blank) was mixed with the freshly FRAP reagent and the mixture was incubated at 37 °C for 15 min. The absorbance at 595 nm was measured on the spectrophotometer. BHT, Trolox, caffeic acid, or gallic acid could be used as a positive control. The IC_50_ of each extract was determined as the concentration that corresponds to 50% of the ferric ion reduction. Values are reported as means ± SD of four independent determinations.

#### 3.4.4. Cupric Reducing Antioxidant Capacity (CUPRAC)

CUPRAC was determined using the method described by Apak et al. [98,99] and Deng et al. [100], with some modifications. Briefly, a 10 mM aqueous copper (II) solution (copper (II) sulfate or copper (II) chloride) and 1 M ammonium acetate buffer solution (pH 7) were previously prepared. The ethanolic solution of neocuproine 7.5 mM was prepared by dissolving 78 mg of this reagent in 50 mL of ethanol. A 20 μg/mL ethanolic gallic acid solution was used as a positive control.

Then, 45 μL of the copper (II) solution, 45 μL of neocuproine, 45 μL of the buffer solution, 50 μL of distilled water, and 45 μL of the sample (45 μL of sample solvent, blank, or 45 μL of positive control, Trolox, or gallic acid) were placed in a 96-well plate. The plate was incubated for 2 min at room temperature in the spectrophotometer and the absorbance at 450 nm was measured. The results were expressed as Trolox equivalent antioxidant capacity (TEAC) and were calculated using the equation reported by Apak et al. [99], with some modification. Values are reported as means ± SD of four independent determinations.

(1) $$\frac{\mathrm{mg}\;\mathrm{Trolox}\;\mathrm{equivalent}}{g\;\mathrm{dry}\;\mathrm{extract}}=\frac{{r\;A}_{f}V_{f}M}{\varepsilon_{\mathrm{TR}}{\;l\;V}_{S}\;C}$$

where

*r*: dilution factor of the measured sample in relation to the starting sample.
*A_f_*: absorbance.
*V_f_*: final volume in well (230 μL).
*M*: molar mass of Trolox (250.29 g/mol).
*ε_TR_*: Trolox molar absorptivity (1.67 × 10^4^ L mol^−1^ cm^−1^).
*l*: optical path length in cm (0.6715 cm).
*V_s_*: sample volume (45 μL).
*C*: concentration of the original solution (mg/mL).

### 3.5. Glucosidase Inhibitory Activity

α-glucosidase inhibitory activity (α-glucosidase type I from baker’s yeast, EC 3.2.1.20) was evaluated following the protocol described by Pistia et al. [101], with slight modifications. Briefly, 20 μL of desired extract was incubated with 20 μL of the enzyme (1 U/mL) for 5 min at 37 °C, and 147 μL of buffer phosphate solution (0.5 M, pH 6.8). After that, the reaction was started with the addition of 13 μL of 0.5 mM of 4-nitrophenyl-α-D-glucopyranoside dissolved in the buffer solution. The extract is replaced by the same volume of ethanol in the control reaction. Acarbose was used as a positive control. The kinetics of the reaction is carried out by measuring every two minutes, for 20 min, at 400 nm. The *p*-nitrophenol obtained was quantified at 400 nm by spectrophotometry, using a calibration curve between 0.05 and 1 mM. The enzyme activity was expressed as mM of *p*-nitrophenol/min. Values are reported as means ± SD of four independent determinations. The inhibition percentage was calculated by the following equation:

(2) $$\%\mathrm{inhibition}=100\times \left(\frac{{\mathrm{Enzyme}\;\mathrm{activity}}_{\mathrm{Control}}-{\mathrm{Enzyme}\;\mathrm{activity}}_{\mathrm{Test}}}{{\mathrm{Enzyme}\;\mathrm{activity}}_{\mathrm{Control}}}\right)$$

### 3.6. Tyrosinase Inhibitory Activity

Tyrosinase inhibitory activity (tyrosinase from mushroom, EC 1.14.18.1) was evaluated following the protocol described by Momtaz et al. [85], with slight modifications. Briefly, 20 μL of desired extract was incubated with 50 μL of the enzyme (500 U/mL) for 5 min at 25 °C, and 30 μL of buffer phosphate solution (50 mM, pH 6.5). After that, the reaction was started with the addition of 120 μL of 2 mM of L-tyrosine. Amino acid and enzyme were dissolved in the same buffer solution. The extract is replaced by the same volume of ethanol in the control reaction. Kojic acid was used as a positive control. The kinetics of the reaction is carried out by measuring every minute, for 30 min, at 475 nm. The enzyme activity was expressed as absorbance units/min of dopachrome. Values are reported as means ± SD of four independent determinations. The inhibition percentage was calculated by the following equation:

$$\%\mathrm{inhibition}=100\times \left(\frac{{\mathrm{Enzyme}\;\mathrm{activity}}_{\mathrm{Control}}-{\mathrm{Enzyme}\;\mathrm{activity}}_{\mathrm{Test}}}{{\mathrm{Enzyme}\;\mathrm{activity}}_{\mathrm{Control}}}\right)$$

### 3.7. Determination of Sun Protector Factor (SPF)

The sun protection factor (SPF) was calculated according to the method described by Mansur et al. [50] and used by other authors [51,52,53]. Briefly, the method consists of measuring the absorbance of the sample between 290 and 320 nm, every 5 nm, and using the following equation: where CF is a correction factor (10), EE (λ) is the erythemal effect spectrum, and I (λ) is the solar intensity spectrum. The EE (λ) × I (λ) values are constant for each wavelength of the interval and they are reported in the literature (Table 4) [51,52,53]. All compounds were tested at approximately 200 μg/mL in 96-well plates. Values are reported as means ± SD of four independent determinations.

$$\mathrm{SPF}=\mathrm{CF}\;\times \overset{320}{\underset{290}{\sum }}\mathrm{EE}\left(\lambda \right)\;\times \;I\;\left(\lambda \right)\;\times \;\mathrm{Abs}\;\left(\lambda \right)$$

### 3.8. Statistical Analysis

All measurements were carried out four times and the results were reported as the mean value ± SD. Statistical analyses were performed using one-way ANOVA and the post hoc comparisons were carried out using Tuckey’s test (Graphpad Prism 6 for windows). Multivariate analysis was performed using principal component analysis (PCA) followed by an analysis of similarity (ANOSIM). Correlations among variables were done using the Pearson’s procedure (*p* < 0.05). The average dissimilarity was performed using the similarity percentages breakdown procedure (SIMPER) (PAST, version 4.03, June 2020).

## 4. Conclusions

This is the first approach to the biochemical and metabolic traits related to the non-enzymatic antioxidant capacity of *Nothofagus* species from the southern Chilean Patagonia. The concentrations of the studied metabolites in the species of the genus *Nothofagus* were very similar to those found in *A. chilensis*. The anthocyanin content observed in *N. antarctica*, *N. pumilio*, and *N. dombeyi* was higher than in *A. chilensis* and *B. microphylla*. The *Nothofagus dombeyi* and *Nothofagus betuloides* antioxidant capacity, measured as DPPH, ABTS, and FRAP, showed statistically similar values to *B. microphylla* and *A. chilensis*. These *Nothofagus* are the species with the best antioxidant capacity, the first being the best of all. Multivariate analysis showed that *B. microphylla* differs from all studied species. Moreover, the *Nothofagus* split into two groups. *N. betuloides* and *N. dombeyi* are the most similar species to *A. chilensis*. Furthermore, some foliar extracts showed an excellent sun protection factor, with *B. microphylla* being the highest. Finally, the five species of the genus *Nothofagus* showed 100% of α-glucosidase inhibitory activity, and three of them exhibit good tyrosinase inhibitory activity. These results make them potential antidiabetic drugs, functional foods, and skin protectors and lightener.

## Figures and Tables

**Table 1:** Analysis of similarity (ANOSIM), R values.

	*A. chilensis*	*N. dombeyi*	*N. betuloides*	*N. nitida*	*N. pumilio*	*N. antarctica*	*B. microphylla*
*A. chilensis*		0.7778	0.8519	1	1	0.9259	1
*N. dombeyi*	0.7778		0.2963	1	1	0.963	1
*N. betuloides*	0.8519	0.2963		1	1	0.9259	1
*N. nitida*	1	1	1		0.5926	0.7778	1
*N. pumilio*	1	1	1	0.5926		0.03704	1
*N. antarctica*	0.9259	0.963	0.9259	0.7778	0.03704		1
*B. microphylla*	1	1	1	1	1	1	

**Table 2:** Sun protection factor of the extracts.

Species	SPF	SD	Extract Concentration in the Assay (μg/mL)	Extract Mass in the Assay (μg)	SPF/mg of Dry Extract
*B. microphylla*	19.32	0.66	179	35.8	539 ± 16
*A. chilensis*	10.03	0.22	189	37.8	266 ± 5
*N. dombeyi*	8.39	0.12	219	43.8	191 ± 2
*N. antarctica*	8.31	0.45	252	50.4	165 ± 5
*N. betuloides*	7.81	0.05	206	41.2	190 ± 1
*N. pumilio*	5.79	0.06	236	47.2	123 ± 1
*N. nitida*	3.10	0.12	190	37.9	82 ± 3

**Table 3:** α-glucosidase and tyrosinase inhibitory activity of plant extracts.

	α-Glucosidase		Tyrosinase		
*Species*	Inhibition (%)	SD	Inhibition (%)	SD	Concentration in the Assay (μg/mL)
*N. antarctica*	100	0	72.7	3.9	84
*N. pumilio*	100	0	68.5	4.2	118
*N. dombeyi*	100	0	40.8	4.5	98
*N. nitida*	100	0	69.3	2.7	95
*N. betuloides*	100	0	18.4	1.7	103
*A. chilensis*	99.0	0.4	31.7	4.8	94
*B. microphylla*	98.4	0.3	52.3	3.6	90
Acarbose	70.3	3.7	-	-	616
Kojic acid	-	-	100	0	45

**Table 4:** Normalized EE (λ) x I (λ) values reported.

λ (nm)	EE (λ) × I (λ)
290	0.0150
295	0.0817
300	0.2874
305	0.3278
310	0.1864
315	0.0839
320	0.0180

## Data Availability

Not applicable.

## Supplementary Materials

The following are available online. Table S1. PCA loading vectors. Table S2. The similarity percentages breakdown procedure (SIMPER). Figure S1. Sampled species.
