# Supplementary material for: Phytochemical Screening and Antioxidant Activity of Seven Native Species Growing in the Forests of Southern Chilean Patagonia
Source: Molecules. 2021 Nov 6;26(21):6722. doi: 10.3390/molecules26216722 (PMC8587661; doi:10.3390/molecules26216722)
Supplement: Supplementary file 1 [file molecules-26-06722-s001.zip › molecules-1422650-supplementary.pdf]

Table S1. PCA loading vectors.

Vectors	PCA1	PCA2
TPC	0.41154	– 0.41358
TFC	0.49299	0.20456
TCC	0.48567	0.18417
THC	0.54696	0.30095
TAC	– 0.0013034	– 0.00025391
DPPH	– 0.030853	0.22122
ABTS	– 0.023584	0.10372
FRAP	– 0.22592	0.77648
CUPRAC	0.00064151	– 0.0010751

Table S2 – Part 1. The similarity percentages breakdown procedure (SIMPER).

Var	Average dissimilarity					
	**1 vs 2**	**1 vs 3**	**1 vs 4**	**1 vs 5**	**1 vs 6**	**1 vs 7**
DPPH	0.2746	0.2589	2.494	2.67	0.9959	0.3311
ABTS	0.1453	0.07301	0.9007	1.196	0.7182	0.07199
FRAP	1.703	0.4618	7.632	7.857	5.55	1.534
CUPRAC	0.006417	0.002167	0.008923	0.009068	0.008525	0.009728
TPC	1.428	2.752	4.738	5.724	5.772	5.587
TFC	0.416	1.082	1.65	0.1523	0.3271	10.06
THC	1.758	1.905	2.688	1.93	1.891	11.61
TCC	4.006	4.594	3.497	0.7693	0.4669	8.265
TAC	0.05383	0.006352	0.002956	0.0306	0.05213	0.01046
OAD*	9.792	11.135	23.611	20.338	15.782	37.479

1: *A. chilensis*; 2: *N. dombeyi*; 3: *N. betuloides*; 4: *N. nitida*; 5: *N. pumilio*; 6: *N. antarctica*; 7: *B. microphylla* (leaf); 8: *B. microphylla* (flower). * OAD: Overall average dissimilarity.

Table S2 – Part 2. The similarity percentages breakdown procedure (SIMPER).

Var	Average dissimilarity								
	**2 vs 3**	**2 vs 4**	**2 vs 5**	**2 vs 6**	**2 vs 7**	**3 vs 4**	**3 vs 5**	**3 vs 6**	**3 vs 7**
DPPH	0.4168	2.749	2.937	1.178	0.4256	2.46	2.651	0.8292	0.1839
ABTS	0.1478	1.073	1.386	0.8895	0.05581	0.9781	1.299	0.784	0.07005
FRAP	1.715	9.402	9.65	7.291	0.8212	8.449	8.696	6.227	1.493
CUPRAC	0.005714	0.01524	0.01543	0.01516	0.006044	0.01152	0.01169	0.01123	0.008999
TPC	3.397	5.302	6.346	6.426	5.593	2.679	3.641	3.593	7.662
TFC	0.7218	2.105	0.3538	0.6913	10.71	2.785	0.9678	1.37	11.33
THC	0.3767	3.051	1.649	1.349	12.22	3.44	2.004	3.593	12.65
TCC	0.5059	0.3277	2.916	3.415	11.16	0.4542	3.433	3.971	11.67
TAC	0.05257	0.0493	0.01779	0.01346	0.04552	0.006068	0.02758	0.05087	0.01486
OAD*	7.339	24.074	25.271	21.27	41.03	21.26	22.73	18.55	45.08

1: *A. chilensis*; 2: *N. dombeyi*; 3: *N. betuloides*; 4: *N. nitida*; 5: *N. pumilio*; 6: *N. antarctica*; 7: *B. microphylla* (leaf); 8: *B. microphylla* (flower). * OAD: Overall average dissimilarity.

Table S2 – Part 3. The similarity percentages breakdown procedure (SIMPER).

Var	Average dissimilarity					
	**4 vs 5**	**4 vs 6**	**4 vs 7**	**5 vs 6**	**5 vs 7**	**6 vs 7**
DPPH	0.5247	1.51	1.367	1.677	1.481	0.3373
ABTS	0.4655	0.4709	0.6646	0.5225	0.8616	0.5361
FRAP	1.686	2.916	6.605	3.107	6.756	5.201
CUPRAC	0.002415	0.002817	0.01528	0.002125	0.0154	0.01524
TPC	1.293	1.176	8.514	0.9638	9.194	9.329
TFC	1.581	1.342	8.471	0.3328	9.644	9.71
THC	1.257	1.64	9.252	0.4356	10.19	10.73
TCC	2.565	2.988	10.23	0.5033	8.417	8.44
TAC	0.02813	0.04804	0.01026	0.01909	0.03058	0.04476
OAD*	9.402	12.09	45.12	7.564	46.59	44.34

1: *A. chilensis*; 2: *N. dombeyi*; 3: *N. betuloides*; 4: *N. nitida*; 5: *N. pumilio*; 6: *N. antarctica*; 7: *B. microphylla* (leaf); 8: *B. microphylla* (flower). * OAD: Overall average dissimilarity.

Figure S1. Sampled species.

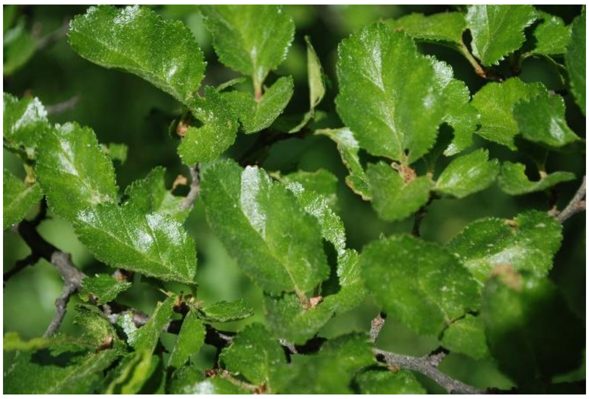

Nothofagus antarctica

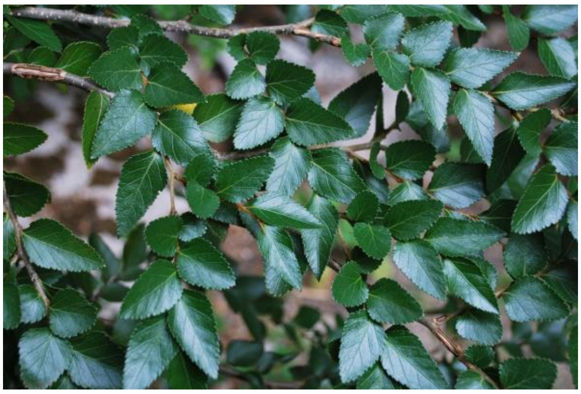

Nothofagus nitida

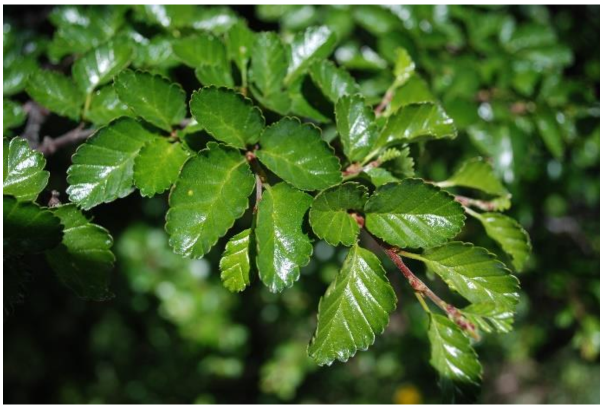

Nothofagus pumilio

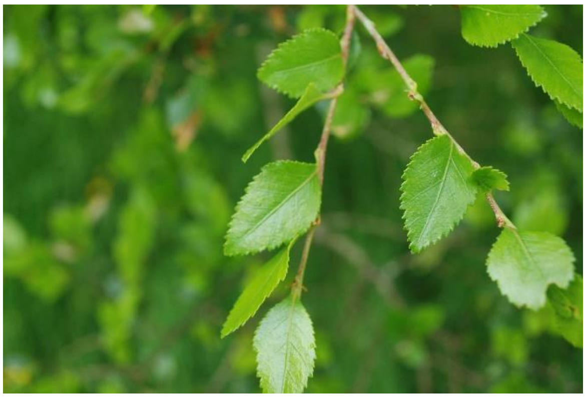

Nothofagus dombeyi

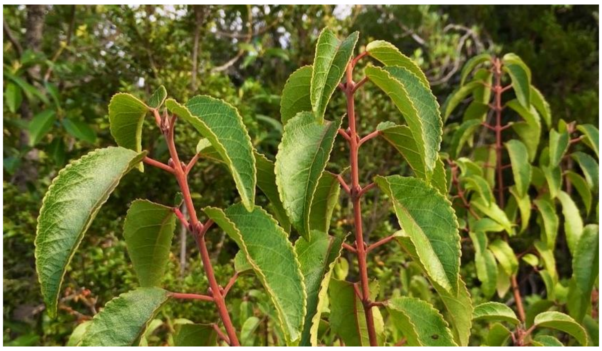

Aristotelia chilensis

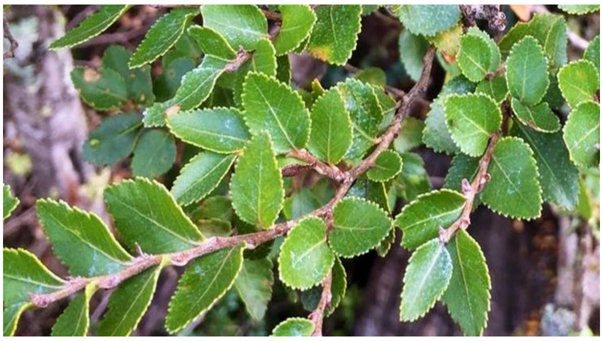

Nothofagus betuloides

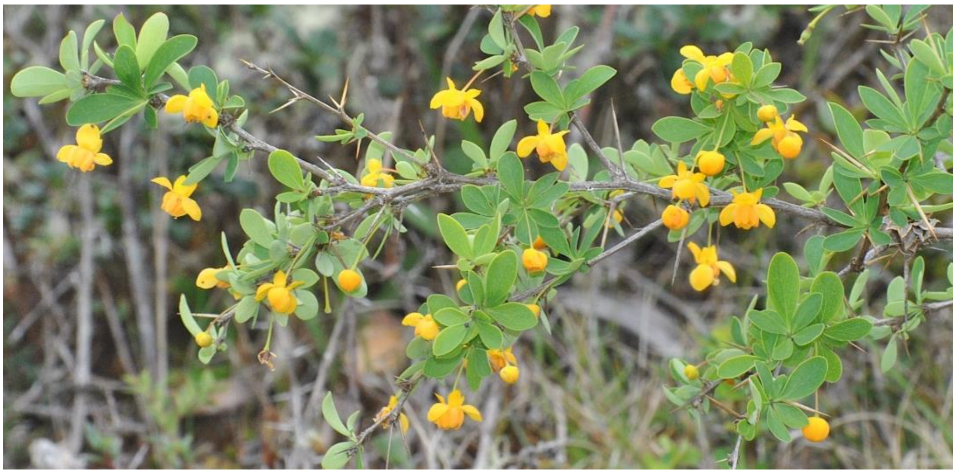

Berberis microphylla
